# An easily overlooked cause of pulmonary arterial hypertension—thiamine deficiency

**DOI:** 10.3389/fnut.2025.1633864

**Published:** 2025-07-31

**Authors:** Qianqian Zhao, Menglin Li, Lina Chen, Fangfang Qiu

**Affiliations:** ^1^Department of Nursing, the Fourth Affiliated Hospital of School of Medicine, and International School of Medicine, International Institutes of Medicine, Zhejiang University, Yiwu, China; ^2^Department of Geriatrics, The Fifth Hospital of Jinhua, Jinhua, China; ^3^Department of Critical Care Medicine, The Fourth Affiliated Hospital of School of Medicine, International School of Medicine, International Institutes of Medicine, Zhejiang University, Yiwu, China

**Keywords:** thiamine, beriberi, pulmonary arterial hypertension, hemodynamics, trace elements

## Abstract

Thiamine deficiency (TD), also known as vitamin B1 deficiency, is an often overlooked potential cause of pulmonary arterial hypertension (PAH). It may contribute to the pathological process of PAH through the deficiency of related coenzymes, thereby inhibiting the citric acid cycle. TD can manifest in various clinical forms, including dry beriberi and Shoshin beriberi, the latter characterized by high-output heart failure and lactic acidosis, which can lead to fatal outcomes if not promptly treated. This article reviews the physiological functions of thiamine, the pathophysiological mechanisms of TD, and its relationship with PAH. It explores the hemodynamic changes and diagnostic challenges of Shoshin beriberi and highlights the potential value of thiamine supplementation therapy. Although large-scale randomized controlled trials are currently lacking, minor studies and case reports suggest that thiamine supplementation may benefit PAH patients. Future high-quality research is needed to clarify the role of thiamine in the treatment of PAH.

## 1 Introduction

Thiamine (vitamin B1), as a crucial coenzyme, plays an essential role in the tricarboxylic acid (TCA) cycle and the pentose phosphate pathway, making it an indispensable component of energy metabolism. Thiamine deficiency (TD) can lead to multisystem dysfunction, with cardiovascular manifestations including high-output heart failure and abnormal peripheral vascular resistance (1). In recent years, numerous studies (2–5) have indicated that TD may contribute to the pathological progression of pulmonary arterial hypertension (PAH) through mechanisms such as mitochondrial dysfunction, enhanced oxidative stress, and endothelial homeostasis imbalance. PAH is a hemodynamic pathological condition characterized by abnormally elevated pulmonary arterial pressure, with high morbidity and mortality rates. This article aims to explore the relationship between thiamine deficiency and PAH, analyze the pathological mechanisms and diagnostic challenges of Shoshin beriberi, and evaluate the potential value of thiamine supplementation therapy, thereby providing new perspectives and treatment strategies for clinical practice.

## 2 Physiological functions of thiamine

Thiamine, discovered in 1925 as the first vitamin, also known as vitamin B1, is typically stored in the body in amounts of only 30 milligrams, with a half-life of 10 to 18 days. It can be depleted within 20 days of insufficient oral intake (6). The ability to biosynthesize thiamine is species-specific. Current research (2) indicates that only plants, microorganisms, and some fungi can biosynthesize thiamine, while humans and other mammals, lacking the relevant enzymatic systems, must rely on exogenous thiamine supply (through dietary intake or synthesis by gut microbiota). Although specific bacteria in the human gut microbiota (such as Bacteroidetes and Actinobacteria) possess the ability to synthesize thiamine (7, 8), their production is limited by factors such as microbial abundance, host metabolic status, and dietary structure, making it challenging to meet the body’s daily requirements (approximately 1.2–1.4 mg/day for adult males and 1.0–1.2 mg/day for adult females). Therefore, humans can obtain sufficient thiamine through a diversified diet, including grains, vegetables, meats, and nuts.

Thiamine is an essential water-soluble vitamin for the human body, playing a critical role in energy metabolism. In living organisms, thiamine primarily exists in three phosphorylated forms: thiamine monophosphate (ThMP), thiamine diphosphate (ThDP), and thiamine triphosphate (ThTP). Among these, ThDP serves as a key coenzyme involved in carbohydrate and amino acid metabolism. Specifically, ThDP is an essential cofactor for several dehydrogenase complexes, including (1) the branched-chain *α*-keto acid dehydrogenase complex, which catalyzes the conversion of branched-chain *α*-keto acids into their corresponding acyl-CoA derivatives; (2) the *α*-ketoglutarate dehydrogenase complex, which mediates the conversion of α-ketoglutarate to succinyl-CoA; (3) the pyruvate dehydrogenase complex, responsible for converting pyruvate into acetyl-CoA. These enzyme complexes play catalytic roles in the TCA cycle (2, 9). A reduction in their activity can limit the supply and cycling of the TCA cycle, leading to oxidative damage, decreased adenosine triphosphate (ATP) synthesis, and cell death. These changes can result in the accumulation of harmful intermediates such as lactate and pyruvate in tissues, subsequently causing organ dysfunction and symptoms of lactic acidosis (9).

## 3 Pathophysiological mechanisms of thiamine deficiency

The primary causes of thiamine deficiency (TD) include insufficient thiamine intake, the effects of anti-nutritional factors, and impaired absorption. Chronic alcoholism, excessive vomiting, starvation, or prolonged use of loop diuretics can all lead to inadequate thiamine intake (10). Additionally, excessive cooking of food can destroy thiamine, while polyphenols in tea and caffeine can interfere with thiamine absorption through competitive inhibition. Notably, thiaminase, found in raw fish, shellfish, and certain nuts, can degrade thiamine molecules. Alcohol can also impair thiamine absorption in the jejunum, a condition that may also occur after bariatric surgery, in patients with chronic diarrhea, or those with other malabsorption syndromes. In developed countries, high-risk populations for thiamine deficiency include individuals with alcohol dependence, severe dietary restrictions, and older people. In recent years, cases of thiamine deficiency have also been reported among incarcerated populations (11).

Thiamine is primarily transported via red blood cells to metabolically active tissues and organs, including the brain, heart, liver, pancreas, muscles, and nervous system. When thiamine deficiency (TD) occurs in these systems, the initial manifestations are metabolic disturbances and severe malnutrition (12). Since myocardial cells require a continuous supply of energy, the disruption of aerobic metabolic pathways caused by TD interrupts the energy supply for cardiac contraction. Additionally, thiamine-dependent nitric oxide synthase further exacerbates circulatory dysfunction, as this enzyme is closely related to pulmonary capillary function in patients.

TD leads to severe physiological and metabolic disturbances, primarily characterized by capillary leakage and systemic vascular resistance reduction due to peripheral vasodilation. This reduction in vascular resistance is initially subtle but, as the condition progresses, can result in decreased diastolic pressure, tachycardia, and hyperdynamic circulation, a condition known as “Shoshin beriberi.” The hemodynamic features of this disease include high-output heart failure associated with slight artery vasodilation, accompanied by increased pulmonary blood flow and elevated left ventricular end-diastolic pressure. Increased pulmonary capillary wedge pressure and elevated pulmonary vascular resistance may represent another important mechanism contributing to PAH, thereby increasing cardiac preload. This increase in preload and myocardial dysfunction can ultimately lead to congestive heart failure, predominantly manifesting as right-sided heart failure. Notably, patients with wet beriberi typically maintain standard or hyperdynamic left ventricular systolic function (13).

## 4 Pathophysiological basis of pulmonary arterial hypertension

PAH is a hemodynamic pathological condition characterized by abnormally elevated pulmonary arterial pressure. It is defined as a mean pulmonary arterial pressure (mPAP) ≥ 20 mmHg at rest, as measured by right heart catheterization (typical value: 14 ± 3 mmHg) (14). This pathological state leads to increased afterload on the right ventricle, resulting in right ventricular hypertrophy, dilation, and ultimately progressing to right heart failure, accompanied by corresponding clinical symptoms. PAH is not an independent disease entity but rather a clinical syndrome caused by various etiologies. Its etiological spectrum is broad, arising either directly from pulmonary vascular pathologies or secondary to cardiac, pulmonary, or systemic diseases. To standardize clinical diagnosis and treatment, the World Health Organization (WHO) systematically established the clinical classification criteria for PAH during the Second World Symposium on PAH held in Evian, France, in 1998. This classification has undergone multiple revisions and remains a critical guideline for clinical practice.

Based on etiology and pathophysiological mechanisms, PAH can be categorized into arterial PAH, PAH associated with left heart disease, hypoxia-related PAH, and others. The pathogenesis of PAH is complex, involving multiple pathophysiological processes (14), including (1) Vasoconstriction: Increased levels of vasoconstrictors such as endothelin-1 lead to pulmonary arterial constriction. (2) Vascular remodeling: Proliferation and migration of pulmonary arterial smooth muscle cells, along with inflammatory cell infiltration, cause thickening of the pulmonary vascular walls. (3) Inflammatory response: Inflammatory cells and cytokines play a significant role in the development and progression of PAH. (4) Oxidative stress: Oxidative stress damages pulmonary vascular endothelial cells and promotes vascular remodeling.

Thiamine is a crucial coenzyme in human energy metabolism, participating in the conversion of pyruvate to acetyl-CoA during glucose metabolism—a key step for entry into the TCA cycle, which provides energy to cells. When thiamine is deficient, this metabolic pathway is disrupted, leading to pyruvate accumulation and increased lactate production. At the same time, the insufficient supply of acetyl-CoA impairs the TCA cycle, reducing cellular energy production and causing a decline in intracellular ATP levels. The ATP deficiency triggers a cascade of pathological effects, including impaired energy supply to cardiomyocytes, which weakens myocardial contractility and compromises cardiac pumping function, ultimately contributing to heart failure. This elevates pulmonary circulation pressure and promotes the development of pulmonary hypertension. Additionally, metabolic dysfunction induces peripheral vasodilation and capillary leakage, increasing venous return and cardiac workload, further exacerbating pulmonary circulatory pressure (15).

During thiamine deficiency, the body experiences increased oxidative stress, leading to elevated levels of reactive oxygen species (ROS) and reactive nitrogen species (RNS). ROS, such as superoxide anions, are highly oxidative and reactive, directly damaging cellular membranes, proteins, DNA, and other biomolecules, thereby disrupting cell structure and function. In pulmonary vascular endothelial cells, ROS inactivate nitric oxide (NO)—a critical vasodilator—resulting in pulmonary vasoconstriction and increased vascular resistance. Additionally, ROS activate signaling pathways such as NF-κB, triggering the release of inflammatory cytokines that further damage pulmonary vascular endothelial cells and promote the progression of pulmonary hypertension (16).

Thiamine deficiency-induced oxidative stress and impaired energy metabolism lead to dysfunction of pulmonary vascular endothelial cells. These compromised endothelial cells lose their ability to properly synthesize and release vasoactive substances, such as nitric oxide and prostacyclin, which play crucial roles in maintaining vascular tone balance and inhibiting vascular smooth muscle cell proliferation. The endothelial dysfunction disrupts the equilibrium between vasoconstriction and vasodilation, resulting in predominant vasoconstriction while simultaneously promoting the proliferation and migration of vascular smooth muscle cells. This process initiates pulmonary vascular remodeling (16).

The metabolic dysfunction and oxidative stress generate various cytokines and growth factors, including platelet-derived growth factor (PDGF) and endothelin-1 (ET-1), which stimulate the proliferation and migration of pulmonary arterial smooth muscle cells. These cells migrate from the vascular media to the intima, where they proliferate, resulting in vascular wall thickening, luminal narrowing, and increased pulmonary vascular resistance (17).

During pulmonary vascular remodeling, fibroblasts are activated and differentiate into myofibroblasts, which synthesize and secrete excessive extracellular matrix components such as collagen. The abnormal collagen deposition within the vascular wall leads to increased stiffness and reduced elasticity, further exacerbating elevated pulmonary vascular resistance and pulmonary hypertension (18).

## 5 Pathological mechanisms and hemodynamic changes in Shoshin beriberi: from metabolic acidosis to acute heart failure

As Shoshin beriberi progresses and becomes more complex, it can develop into malignant, fulminant, and advanced wet beriberi. Its typical features include severe metabolic acidosis, biventricular failure, increased cardiac output, reduced peripheral vascular resistance, and skin cyanosis, which may rapidly lead to death. Clinically, patients often present with severe shock-like conditions, such as hypotension, lactic acidosis, and tachycardia. However, the condition can be rapidly reversed upon oral administration of thiamine, leading some scholars to describe Shoshin beriberi as “a rapidly curable hemodynamic disaster” (19).

These hemodynamic changes significantly increase the burden on the right ventricle, potentially leading to right ventricular hypertrophy or even failure. When pulmonary arterial pressure rises sharply, right atrial and/or right ventricular pressures increase rapidly, impeding venous return and causing a sharp rise in central venous pressure (CVP). Simultaneously, dilation of the valve annulus leads to tricuspid regurgitation, further increasing proper ventricular preload and causing right ventricular enlargement. The enlarged right ventricle compresses the left ventricle via the interventricular septum, impairing its filling function and leading to elevated left atrial pressure, which further increases pulmonary arterial pressure. This series of changes further reduces right and left heart perfusion exacerbates right heart dysfunction and ultimately creates a self-perpetuating vicious cycle.

At the same time, the decline in cardiac output leads to increased venous return pressure in organs and reduced adequate perfusion pressure in peripheral tissues, resulting in tissue and organ ischemia and hypoxia. This pathological state not only causes severe and irreversible damage to the body but also further promotes lactate production (20).

**Figure 1:**
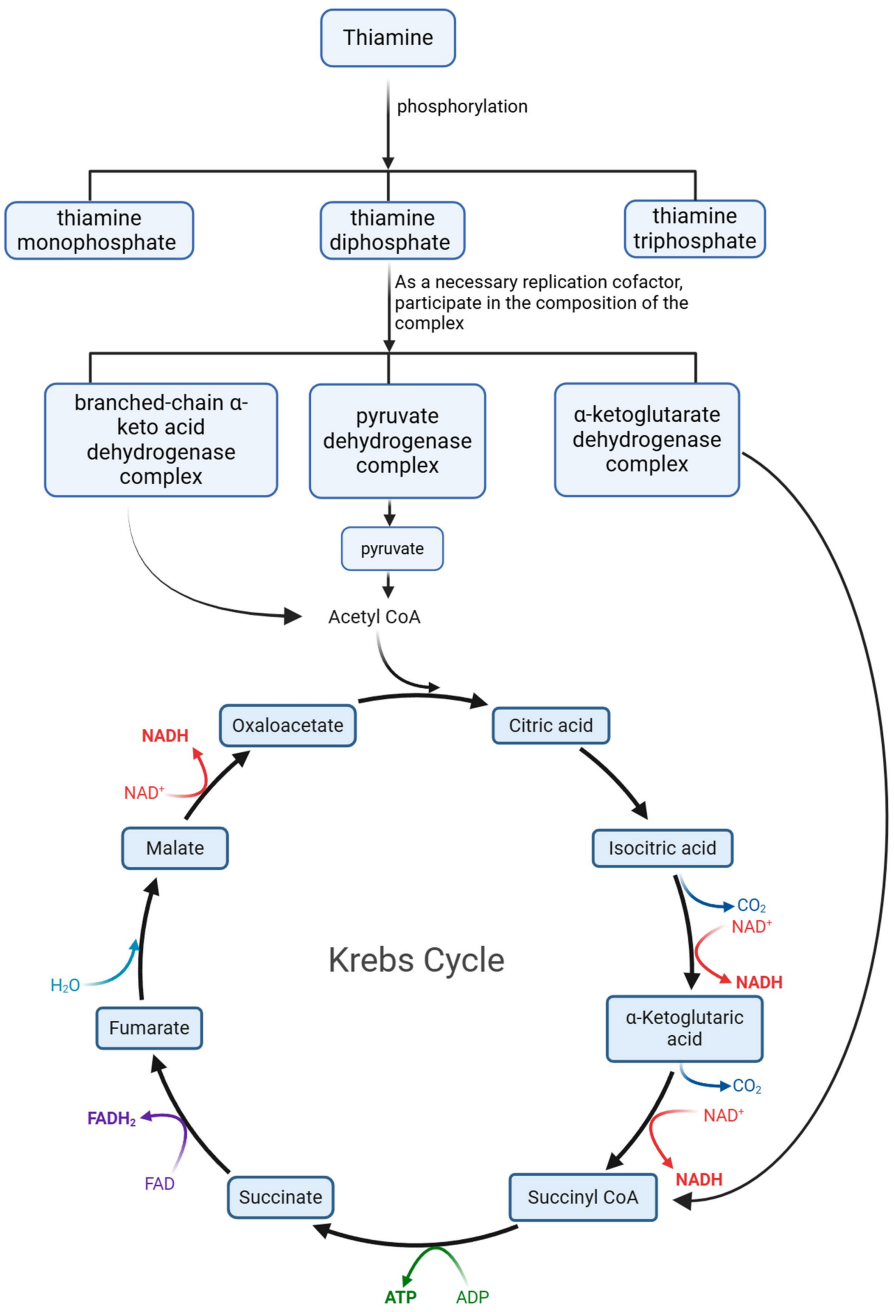
During the metabolism of thiamine in the body, its metabolite Acetyl CoA participates in the Krebs Cycle as an essential product. Acetyl CoA, Acetyl Co-enzyme A; CO_2_, Carbon dioxide; ATP, adenosine triphosphate; NAD+, Nicotinamide Adenine Dinucleotide; NADH, Nicotinamide Adenine Dinucleotide Hydride; ADP, Adenosine Diphosphate; ATP, Adenosine Triphosphate; FAD, Flavin Adenine Dinucleotide; FADH2, Flavin Adenine Dinucleotide in its reduced form; H_2_O, Water.

The mechanism by which thiamine deficiency leads to hyperlactatemia in wet beriberi may be related to the depletion of adenosine triphosphate (ATP) in myocardial cells and the increased production of adenosine. When myocardial cells cannot generate sufficient ATP, adenosine monophosphate (AMP) accumulates and is converted into adenosine. As intracellular adenosine production increases, it accumulates within the cell and is released into the plasma through nucleoside transporters in the cell membrane. Due to thiamine deficiency, the related enzymatic reactions are impaired, causing the citric acid cycle to be blocked and preventing pyruvate from being converted into acetyl-CoA, thereby inhibiting ATP production. This process triggers cellular acidosis and increases intracellular levels of free fatty acids (Figure 1).

**Figure 2:**
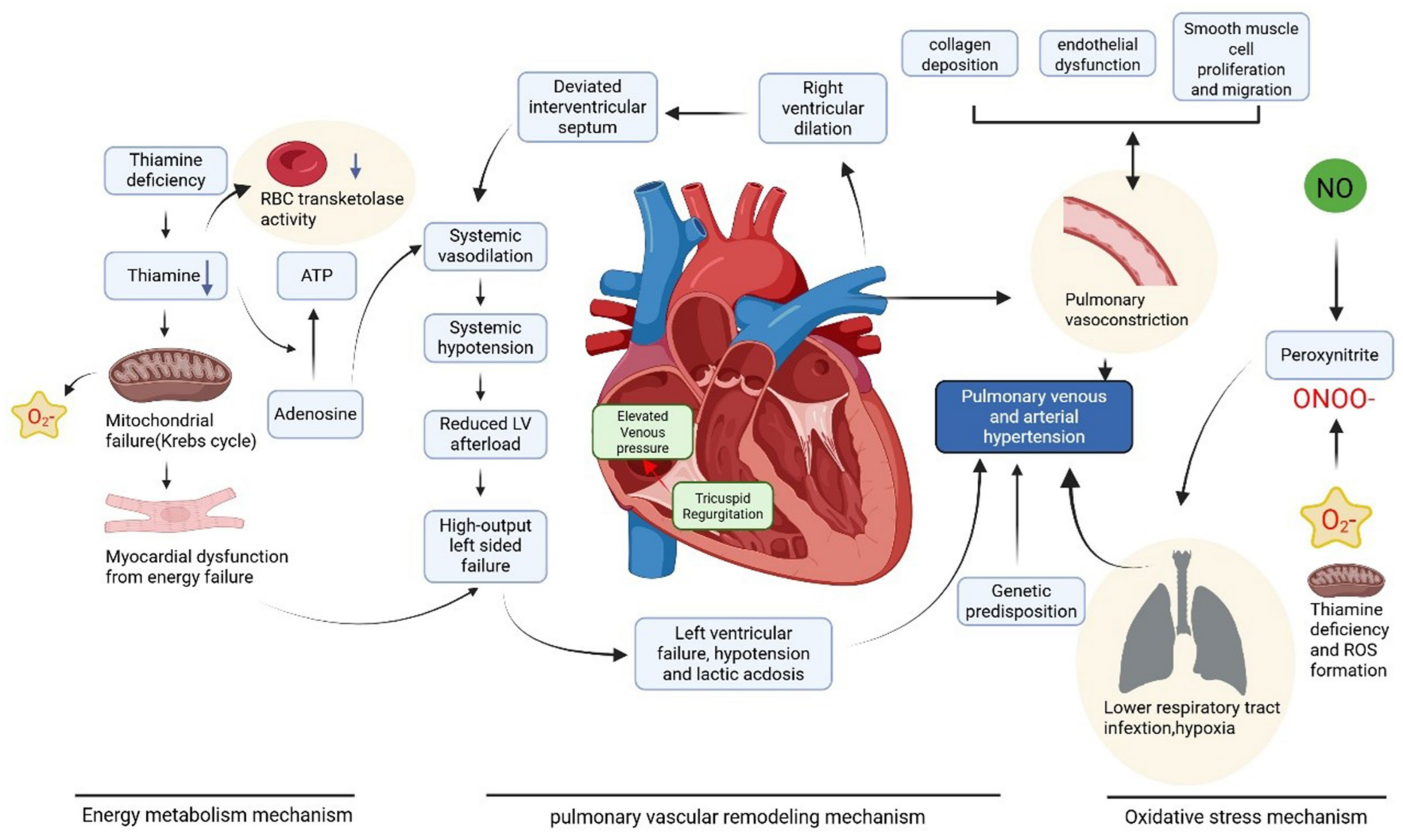
Thiamine deficiency leads to pulmonary hypertension in wet beriberi through multiple mechanisms including impaired energy metabolism, increased oxidative stress, and pulmonary vascular remodeling. Created with BioRender.com.

The lack of ATP forces the body to upregulate glycolysis and mobilize fat resources (such as subcutaneous fat) to meet energy demands. Although thiamine-dependent reactions remain impaired, the utilization of fat resources not only provides energy but also generates ketone bodies. Ketone bodies can be used for acetyl-CoA synthesis in extrahepatic tissues. However, due to increased pyruvate production and reduced utilization, its accumulation promotes increased lactate production, leading to lactic acidosis (21). The buildup of pyruvate and lactate reduces peripheral vascular resistance and increases venous return, thereby increasing cardiac preload. The combination of increased cardiac preload and impaired energy metabolism in myocardial cells caused by thiamine deficiency collectively triggers acute congestive heart failure (Figure 2).

Patients exhibit severe hemodynamic disturbances and acute heart failure, with hemodynamic monitoring showing increased cardiac output and reduced peripheral circulatory resistance. This “high-output, low-resistance” hemodynamic profile differs from septic shock and is a hallmark of Shoshin beriberi. However, diagnosis cannot rely solely on this characteristic hemodynamic outcome; it also requires ruling out infections and confirming the presence of thiamine deficiency, among other factors (6).

## 6 Clinical manifestations and diagnostic challenges of thiamine deficiency: from beriberi subtypes to evaluation and monitoring of pulmonary arterial hypertension

Thiamine deficiency (TD) can manifest in four typical clinical forms: peripheral polyneuropathy, anorexia, and muscle weakness (dry beriberi), as well as high-output heart failure with congestive symptoms (Shoshin beriberi) (22). In the diagnosis of beriberi, obtaining a detailed patient history is crucial. If a patient presents with unexplained lactic acidosis, heart failure symptoms, and a history of chronic malnutrition or alcoholism, TD should be highly suspected. Although the diagnostic process may seem straightforward, in practice, diagnosing cardiac beriberi is challenging based solely on routine tests unless blood thiamine levels are measured immediately upon admission.

In clinical practice, the diagnosis of TD is often delayed because it primarily relies on clinical manifestations and dietary history rather than direct blood or urine tests. Even when such tests are performed, samples usually need to be sent to external laboratories, resulting in long waiting times for results. Among the few laboratories capable of performing these tests, the primary assays include serum or urine thiamine levels (normal reference range: 8–30 nmol/L). At the same time, erythrocyte or erythrocyte transketolase activity (ETKA) testing is less commonly available. However, these methods have limitations. For example, even if thiamine stores in the body are significantly depleted, serum and urine thiamine levels may still appear normal if the patient has recently consumed thiamine-rich foods. In contrast, ETKA testing provides a more accurate reflection of thiamine reserves (23).

In the context of PAH, if the mean pulmonary arterial pressure (mPAP) at rest is ≥25 mmHg, pulmonary capillary wedge pressure (PCWP) is <15 mmHg and pulmonary vascular resistance (PVR) is >3 Wood units, PAH should be considered, provided that left heart disease, chronic lung disease, or venous thromboembolism have been ruled out. In critically ill patients with acute PAH, circulatory failure is often present, making pulmonary arterial pressure monitoring a critical component of hemodynamic assessment. Currently, the primary methods for monitoring pulmonary arterial pressure include crucial care ultrasound and pulmonary artery catheterization (24).

For patients with acute circulatory failure, central venous pressure (CVP) monitoring, continuous arterial blood pressure monitoring, and critical care ultrasound are fundamental rapid assessment tools. The use of a pulmonary artery catheter can quickly confirm a high cardiac output state, which is particularly valuable in cases of rapid clinical deterioration. With appropriate intravenous thiamine supplementation, patients can show significant improvement within hours (25). A case report demonstrated that Swan-Ganz catheterization revealed a pulmonary artery pressure of 48/17 (30) mmHg, a pulmonary capillary wedge pressure of 11 mmHg, a cardiac index of 2.47 L/min/m^2^, and a pulmonary vascular resistance of 5.2 Wood units. After 10 days of thiamine supplementation, follow-up hemodynamic measurements showed a reduction in pulmonary artery pressure to 27/8 (16) mmHg, a pulmonary capillary wedge pressure of 7 mmHg, an increase in cardiac index to 2.94 L/min/m^2^, and a decrease in pulmonary vascular resistance to 2.6 Wood units (10).

Although Swan-Ganz catheterization is the gold standard for diagnosing PAH, its complexity and associated risks make it unsuitable for early screening. In contrast, echocardiography is non-invasive and easy to perform. It can directly estimate pulmonary artery pressure, aiding in the diagnosis of PAH while also assessing disease severity, treatment efficacy, and prognosis. Echocardiography primarily uses Doppler flow imaging and spectral Doppler techniques to estimate pulmonary artery pressure (24). During bedside cardiac ultrasound, if right ventricular dilation or severe tricuspid regurgitation is observed, further measurement of pulmonary artery systolic pressure (PASP) is necessary to screen for PAH. For example, the author previously reported a case of Shoshin beriberi in which echocardiography revealed right heart enlargement, with a correct ventricular end-diastolic diameter of approximately 34 mm, right atrial size of 56 × 53 mm, a left ventricular ejection fraction (LVEF) of 60%, moderate tricuspid regurgitation with a peak velocity (Vmax) of 3.4 m/s, and a correct ventricular systolic pressure (RVSP) of 66 mmHg, consistent with moderate PAH (26).

Although echocardiography offers the advantages of simplicity, speed, and non-invasiveness in screening for PAH associated with Shoshin beriberi, its typical findings are limited to low systemic vascular resistance (SVR) and high cardiac output (CO) combined with PAH. However, many patients may present with non-specific imaging features, such as ST-segment elevation myocardial infarction (STEMI), non-ST-segment elevation myocardial infarction (NSTEMI), or pericardial effusion, showing only right ventricular enlargement with or without functional impairment and valvular regurgitation. These findings are similar to those of other types of dilated cardiomyopathy and can easily be missed. Therefore, while echocardiography cannot be used alone to diagnose cardiac beriberi, it remains the preferred imaging method for the initial and ongoing evaluation of various cardiomyopathies.

Due to the relative rarity of thiamine deficiency in critically ill patients and its lack of specific clinical manifestations, wet beriberi is often misdiagnosed or overlooked (27). Therefore, in diagnosing Shoshin beriberi, a comprehensive approach combining detailed history-taking, physical examination, and imaging techniques such as ultrasound and Swan-Ganz catheterization is essential. Integrating these findings enables early and accurate diagnosis, providing a critical basis for timely and appropriate treatment. Reported cases were are collected in Table 1 of wet beriberi disease in the past 5 years.

**Table 1:** Reported cases of wet beriberi disease in the past 5 years.

Category	Case 1 (31)	Case 2 (26)	Case 3 (15)	Case 4 (32)	Case 5 (28)
Author	Pache-Wannaz L	Fangfang Qiu	Tomonari Moriizumi	Satoshi Kurisu	Yi-Hsin Hung
Reported year	2025	2024	2024	2024	2023
Age and gender	7 M	39 M	50 M	83 M	47 M
Underlying disease	small stature,BMI 14.9 kg/m^2^, autistic features with extremely selective behavior concerning food	Depression patients	Schizophrenia and diabetes	hypertension	hypertension and alcohol use disorder
Causes	Not documented	Unbalanced diet	Unbalanced diet	Oral diuretics, excessive rice consumption	Monotonous carbohydrate diet for 6 months
Main symptoms and signs	General fatigue and peripheral edema for 48 h	Fatigue for 3 months, chest tightness for half a day, The jugular veins are distended and the whole body is pitting edema	Palpitations and progressive shortness of breath for more than 3 weeks	Excessive pitting edema in the lower extremities, Scrotal and lower limb edema for 3 months	engorged jugular vein and accentuated first heart sound, Auscultation revealed bilateral basal crackles, Pitting oedema was prominent in both legs.
Initial heart rate (bpm)	111	141	120	80	101
Initial blood pressure(mmHg)	116/88	88/52	77/64	144/72	73/38
Electrocardiogram and cardiac ultrasound	right bundle branch with rsR’ in V1-V2, mean pulmonary arterial pressure of 53 mmHg with tricupsid regurgitation allowing a measure of right ventricular pressure of 98 mmHg	Sinus tachycardia, the right ventricle was severely dilated, compressing the left heart and forming a D-shaped sign, The indirect pulmonary artery pressure was 66 mmHg	right-axial deviation, and no pulmonary P wave, right-ventricular enlargement, right-ventricular systolic pressure of 54 mm Hg	no significant abnormalities except for flat T-waves in leads V5-V6, left ventricular (LV) hyperkinesia with an ejection fraction of 72%, early-to-late mitral peak flow velocity ratio of 1:1, peak tricuspid regurgitation velocity of 3.0 m/s	sinus tachycardia without ST-T abnormalities, hyperkinetic left ventricular ejection fraction (LVEF) of 70%, pulmonary artery pressure of 50 mmHg
Hemodynamics evaluation using a pulmonary artery catheter	Not documented	Not documented	mean PAP of 40 mm Hg, cardiac output of 3.92 L/min, a cardiac index of 2.05 L/min/m^2^, pulmonary vascular resistance (PVR) of 612 dynes/s/cm^5^	high CO and low SVR, CO of 8.27 L/min, SVR of 813 dyne/s/cm^5^, mean pulmonary capillary wedge pressure of 15 mmHg, and mean right atrial pressure of 9 mmHg	cardiac output was 11.5 L/min, cardiac index was 5.4 L/min/m2, and systemic venous resistance index was 1,013 dynes/s/cm^5^/m^2^
Initial thiamine concentration	39.6 nmol/L	0.13 ng/mL	2.4 μg/dL	23 ng/mL	Not documented
Drugs and dosage, route of administration	Thiamine 32.5 mg/day IV,multi vitamin	Thiamine 100 mg/day, IM	Thiamine 200 mg/day IV	Thiamine 100 mg/day IV	Thiamine 200 mg IV
Treatment effects and outcomes	48 h after significant improvement of right ventricular function, Oxygen, diuretics, and milrinone were progressively weaned within 3 days	On the third day, the pulmonary artery pressure dropped to 43 mmHg, blood pressure improved, vasoactive drugs were discontinued, and the right heart was significantly smaller, ECMO was successfully weaned on day 4, Transfer out of ICU on the 10th day	blood pressure improved substantially within a few hours, and norepinephrine was discontinued after 12 h, On day 24, right heart catheterization demonstrated normal hemodynamics	30 min after administration SVR dropped rapidly, systemic edema improved after 2 days and completely disappeared after 6 days	Lactic acidosis resolved in 24 h, Haemodynamic indices improved in 24 h, Urine output increased in 48 h

Category	Case6 (28)	Case 7 (10)	Case 8 (33)	Case9 (34)	Case 10 (35)
Author	Yi-Hsin Hung	Junichi Shibayama	Tianliang Ma	Govind K	Berry KL
Reported year	2023	2022	2022	2022	2021
Age and gender	20 M	67F	40 M	54F	74 M
Underlying disease	porencephaly and colpocephaly associated with mental retardation	Partial gastrectomy 31 years ago, colon cancer resection 9 years ago, pulmonary hypertension for 1 year	Alcoholism, malnutrition	Not documented	chronic alcohol, obesity BMI 34.3 kg/m^2^
Causes	A diet consisting mainly of rice and fish	Long-term oral diuretics for lower extremity edema	Not documented	Not documented	Not documented
Main symptoms and signs	progressive exertional dyspnoea for 1 week, The jugular vein was engorged, revealed regular heart rate without murmurs or extra heart sounds	Leg edema for several months, dyspnea during exercise, distended jugular veins, increased second heart sound, and pitting edema in both lower extremities	recurrent chest tightness and lower extremity edema	five-day of right lower-quadrant abdominal pain,mild bilateral parotidomegaly, tenderness was elicited in the right lower quadrant, Other systems were normal	2-month history of progressive exertional dyspnoea and weight gain
Initial heart rate (bpm)	120	89	120	86	112
Initial blood pressure (mmHg)	87/47	97/64	96/48	86/54	177/56
Electrocardiogram and cardiac ultrasound	revealed preserved LVEF (50%) with 40 mmHg systolic pulmonary artery pressure, paradoxical interventricular septal motion was observed along with a D-shaped left ventricle and dilated right heart chambers	sinus rhythm and RSR’ pattern in V1, the size of the right ventricle was enlarged,D-shaped left ventricular cavity	LVEF of 50%	ST-segment elevation in leads V3–V6 with reciprocal ST-segment depression in aVR, inferior vena cava was collapsed	dilated inferior vena cava raising the suspicion for high-output heart failure
Hemodynamics evaluation using a pulmonary artery catheter	high output heart failure, 9.9 L/min cardiac output, 6.9 L/min/m2 cardiac index, 15.0 mmHg pulmonary artery wedge pressure, and 564.0 dynes/s/cm^5^/m^2^ systemic	pulmonary artery pressure of 48/17 (30) mmHg, cardiac index of 2.47 L/min/m^2^, pulmonary vascular resistance of 5.2 Wood units	Measured by pulse indicator continuous cardiac output Normal cardiac index (3.5 L/min/m^2^) Low systemic vascular resistance (463 dynes/s/cm^5^)	Not documented	The pulmonary artery pressure was 100 mmHg, high cardiac output (12.3 L/min by thermodilution) and low systemic vascular resistance (587 dynes/s/cm^5^)
Initial thiamine concentration	Not documented	10 ng/mL	0.13 ng/mL	Not documented	39 nmol/L
Drugs and dosage, route of administration	Thiamine 100 mg/day IV	Thiamine 100 mg/day IV	Thiamine 200 mg/day IM	Thiamine 300 mg/day IV	Thiamine 100 mg/day IV
Treatment effects and outcomes	Off vasopressor in 8 h, Completely recovered from multiple organ failures in 2 days	After thiamine administration, urine output increased and vasoactive drugs were discontinued, after 18 day pulmonary pressure decrease to 27/8 (16) mmHg,discharged on day 22	Narrowing of pulse pressure by 40 mmHg in 28 weeks, Symptoms completely resolved	Lactic acidosis resolved in 27 h, liver function improved in 72 h, ST-segment resolution	Alcohol abstinence and thiamine replacement therapy resulted in narrowing of the pulse pressure by nearly 40 mm Hg over 28 weeks

## 7 The potential value of thiamine supplementation therapy

Shoshin beriberi is described as a rapidly progressing hemodynamic catastrophe, often accompanied by multi-organ failure. Due to its non-specific clinical manifestations and rapid deterioration, delayed treatment can lead to fatal outcomes. Therefore, a timely diagnosis of Shoshin beriberi is critical. Currently, the only curative therapy for Shoshin beriberi is the rapid intravenous administration of thiamine, which can significantly improve hemodynamic parameters within minutes to hours. Even in cases where thiamine deficiency is highly suspected, intravenous thiamine (100–500 mg) can serve as a diagnostic and therapeutic intervention, rapidly reversing severe PAH and lactic acidosis (22).

However, there is no consensus on the optimal dose, formulation, duration, or frequency of thiamine administration. Pharmacokinetic studies indicate that the blood half-life of free thiamine is only 96 min. Although large-scale randomized controlled trials in PAH patients are lacking, several small studies and case reports suggest that thiamine supplementation may have potential benefits for PAH patients. George (9) reported four cases of severe Shoshin beriberi, all presenting with severe lactic acidosis and refractory cardiovascular collapse. These patients received intravenous thiamine at doses ranging from 100 mg to 200 mg, and their hemodynamics improved significantly within hours to 48 h. The authors emphasized that unexplained refractory lactic acidosis should raise suspicion for wet beriberi, prompting immediate empirical thiamine supplementation. This treatment not only rapidly corrects hemodynamic disturbances but also significantly improves prognosis.

The diverse and non-specific clinical manifestations of Shoshin beriberi often lead to diagnostic delays. Yi-Hsin (28) reported two cases and reviewed 19 cases of wet beriberi reported over the past decade, highlighting that early thiamine administration is key to diagnosis and treatment. Once wet beriberi is suspected, intravenous thiamine (typically 100–500 mg) should be administered immediately. Following thiamine supplementation, lactic acidosis and hemodynamic instability often improve significantly or resolve entirely within hours. Most cases continue to receive maintenance therapy with 100 mg of thiamine daily for at least 2 weeks after initial treatment. Clinicians should prioritize thiamine supplementation as the first-line treatment for suspected cases rather than relying solely on laboratory confirmation.

In clinical practice, the dosage and administration route of thiamine should be adjusted based on whether the patient is in a life-threatening condition. For stable patients, oral administration can be considered for follow-up treatment. Additionally, for patients with alcoholic beriberi, the dose should be appropriately increased to ensure therapeutic efficacy due to their heightened thiamine requirements.

In cases of acute decompensation with rapidly progressing right ventricular failure, PAH is a condition with high morbidity and mortality. For circulatory failure caused by hemodynamic instability, it is essential to actively identify reversible causes to prevent severe PAH outcomes while simultaneously maintaining circulatory function. Conservative treatment strategies typically include the use of vasoactive drugs to maintain tissue perfusion, fluid restriction to reduce right heart load, and measures to decrease right ventricular volume overload. However, when the condition deteriorates rapidly, and pharmacological interventions fail to take effect promptly, conventional medical approaches often fall short. In such scenarios, veno-arterial extracorporeal membrane oxygenation (VA-ECMO) can serve as a salvage therapy for high-risk acute patients, providing critical time for rescuing those with refractory PAH (29).

VA-ECMO, as a cardiopulmonary mechanical support device, can effectively reduce right ventricular overload, improve hemodynamic status, and restore tissue oxygenation. Typically, an ECMO blood flow rate of 2.5–4 L/min is sufficient to meet systemic perfusion needs while effectively unloading the right ventricle and avoiding increased left ventricular afterload (30). For example, the author previously reported a case of cardiac arrest due to unexplained PAH. After excluding conditions such as pulmonary embolism, the patient’s hemodynamics improved rapidly with ECMO combined with thiamine therapy, and pulmonary artery pressure decreased significantly from 60 mmHg to normal levels, ultimately saving the patient’s life (26). Currently, research on such combination therapies is minimal. Future studies should expand sample sizes and conduct high-quality, multicenter clinical trials to validate their efficacy and optimize treatment protocols.

## 8 Conclusion

Thiamine deficiency (TD) is an often overlooked potential cause of PAH. Through complex pathophysiological mechanisms, TD leads to high-output heart failure and lactic acidosis, particularly evident in Shoshin beriberi. Diagnosing TD is challenging and often delayed due to its non-specific clinical manifestations. Thiamine supplementation therapy, especially intravenous administration, can rapidly reverse the condition and significantly improve hemodynamic parameters. Although large-scale randomized controlled trials are currently lacking, existing studies suggest that thiamine supplementation may offer potential benefits for PAH patients. Future high-quality, multicenter research is needed to clarify the role of thiamine in PAH treatment and to optimize therapeutic protocols. Integrating detailed history-taking, physical examination, advanced hemodynamic monitoring techniques, early diagnosis, and timely treatment of TD-related PAH can improve patient outcomes and reduce mortality.
